# Calcium channel blocker induced gingival enlargement following implant placement in a fibula free flap reconstruction of the mandible: a case report

**DOI:** 10.1186/s40729-020-00242-6

**Published:** 2020-08-18

**Authors:** Henry Quach, Arijit Ray-Chaudhuri

**Affiliations:** Department of Restorative Dentistry, Royal Sussex County Hospital, Brighton, UK

**Keywords:** Gingival enlargement, Calcium channel blocker, Amlodipine, Implant

## Abstract

**Background:**

Gingival tissue enlargement is a common side effect of antiepileptic medications (e.g. phenytoin and sodium valproate), immunosuppressing drugs (e.g. cyclosporine) and calcium channel blockers (e.g. nifedipine, verapamil, amlodipine) (Murakami et al. 2018, Clin Periodontol 45:S17–S27, 2018). The clinical and histological appearances of lesions caused by these drugs are indistinguishable from one another (Murakami et al. 2018, Clin Periodontol 45:S17–S27, 2018). Drug-induced gingival enlargement is rarely seen in edentulous patients.

**Case presentation:**

This case presents a 72-year-old female with a history of squamous cell carcinoma of the floor of the mouth treated with surgical excision and fibula-free flap reconstruction. Following the uncovering of osseointegrated implants placed in the fibular-free flap, the patient developed gingival enlargement of the floor of the mouth. Cessation of amlodipine and switching to an alternative medication lead to a resolution of the enlarged tissue.

**Conclusions:**

This case illustrates that gingival enlargement can occur around dental implants, most notably in rehabilitation cases in patients who have had head and neck cancer. Clinicians should be aware of the risk of gingival enlargement in hypertensive patients taking calcium channel blockers prior to implant placement.

## Background

Drug-induced gingival enlargement around natural teeth in patients on calcium channel blocker (CCB) therapy is widely reported in the literature, but fewer reports exist for effects of CCBs on the gingivae around dental implants. It was first reported in 1984 by Lederman et al. [1] and subsequently reported prevalence range from 14 [2] to 83% [3]. Nifedipine is the most commonly associated drug [4] with the prevalence lower for amlodipine [5] or verapamil [6]. Amlodipine belongs to the dihydropyridine class of CCBs along with nifedipine [5]. CCBs are the eighth most prescribed drug in the USA, and the most frequently prescribed CCB is amlodipine [7].

CCBs prevent calcium ion influx by binding to L-type calcium channels on vascular smooth muscles. This causes relaxation and vasodilation and reduction in heart rate. This in turn decreases systemic vascular resistance which as a result reduces arterial blood pressure [8]. CCBs are widely used to manage hypertension, angina and cardiac arrythmias.

Gingival enlargement can present as an increased gingival mass and volume. It can range from mild to severe enlargement of papillary or marginal gingival tissues. It more commonly affects the anterior teeth than the posterior teeth and the buccal gingivae than the lingual/palatal gingivae [9, 10]. The enlargement can cause aesthetic and functional issues as well as harbour bacterial biofilm that can lead to periodontal disease.

## Case presentation

### Patient description

The patient is a 72-year-old Caucasian female with history of T4 N0 M0 squamous cell carcinoma (SCC) of the right floor of mouth and mandible.

### Case history

**Fig. 1:**
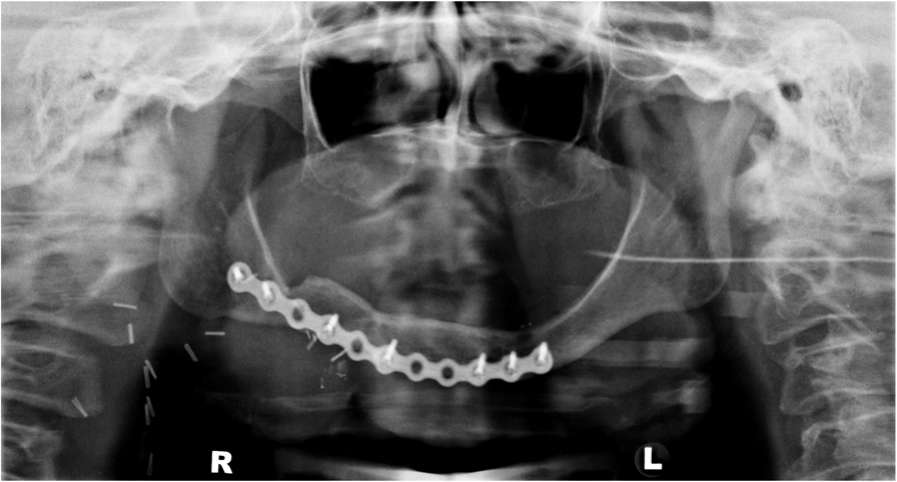
Panoramic radiograph showing segmental mandibulectomy and reconstruction

The patient had a right segmental mandibulectomy and fibula-free flap reconstruction 4 years prior to the events of this case report (Fig. 1). Three years following reconstructive surgery, the patient received restorative dental treatment in the form of mandibular dental implants to support an implant retained denture. The implant placement was carried out without incident.

### Presentation

**Fig. 2:**
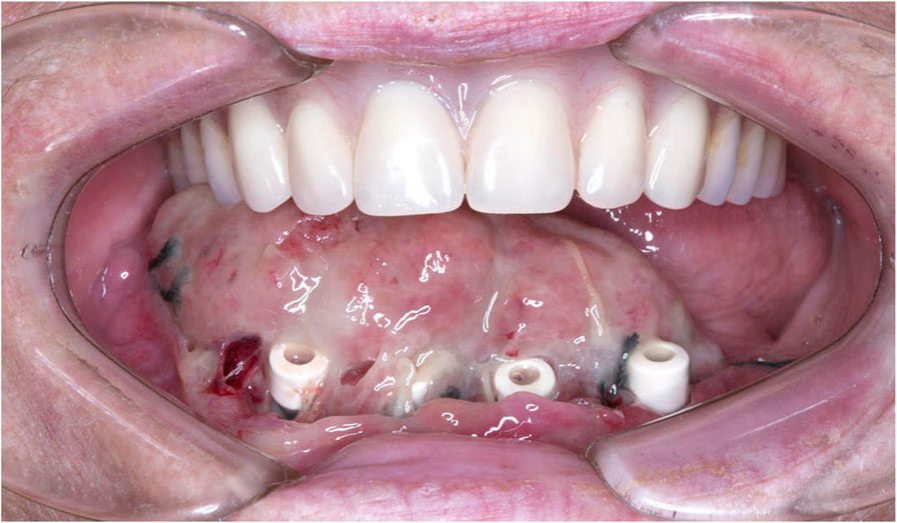
After implant exposure, placement of healing abutments and soft tissue surgery around the dental implants (all done simultaneously). Extensive gingival enlargement of the floor of mouth and lingual gingival tissue

The patient presented with extensive gingival enlargement in the floor of the mouth and lingual gingival tissues (Fig. 2). The firm mass extended bilaterally and partially covered the healing abutments of the implants. The buccal gingivae around the implants were not as severely affected. As the mass presented in the same region as the previous SCC, a biopsy was arranged urgently.

**Fig. 3:**
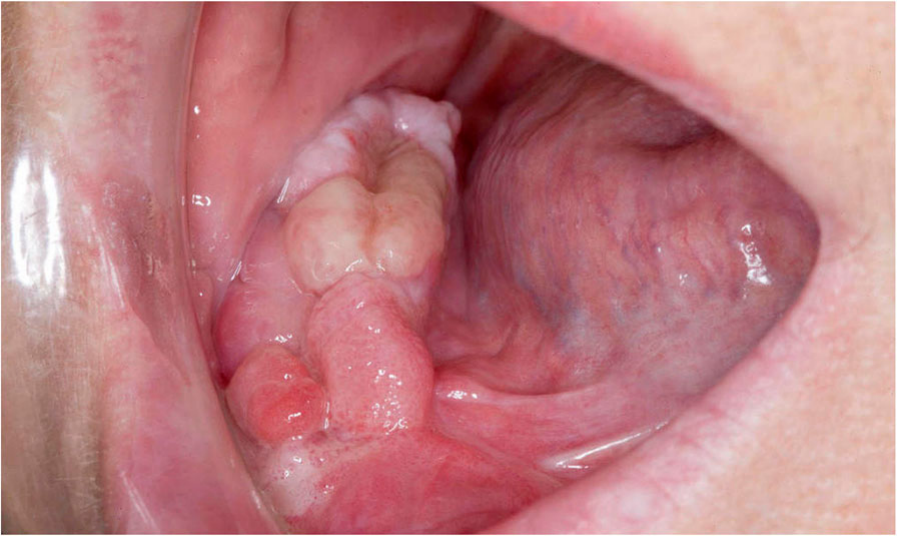
Firm nodular gingival enlargement over the mandibular ridge

The initial overgrowth was subsequently excised under local anaesthetic which leads to a recurrence 4 months later. This recurrence presented as a firm nodular enlargement over the mandibular ridge (Fig. 3). This was also subsequently biopsied to rule out malignancy.

### Results of pathological tests and other investigations

**Fig. 4:**
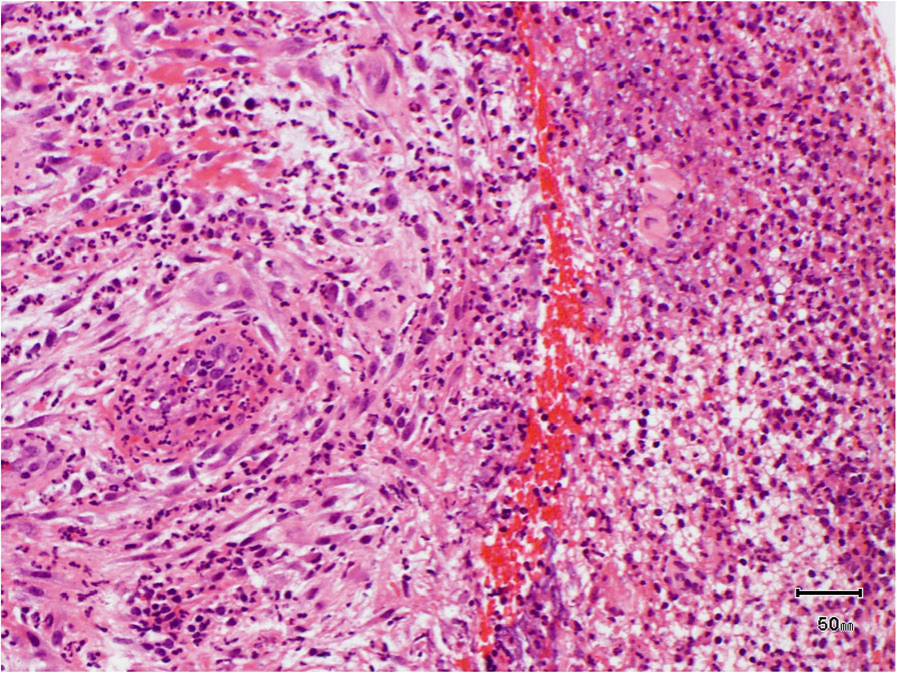
Biopsy floor of the mouth (AE in A1, × 20 magnification)

The patient underwent a series of biopsies to determine the cause for the gingival enlargement. An incisional biopsy was taken from the floor of the mouth (Fig. 4). The floor of mouth biopsy showed mucosa with overlying fibrin and neutrophil polymorphs. The underlying stroma contained a proliferation of thin-walled vessels and fibrosis and neutrophil polymorphs permeating through the depth of the biopsy. In particular, there was no convincing evidence of residual squamous cell carcinoma either morphologically or on immunohistochemistry. This biopsy came to the conclusion of granulation tissue with inflammation. Gingival enlargement is characterised by excess extracellular matrix proteins, non-collagenous proteins and chronic inflammatory infiltrate dominated by plasma cells.

The second biopsy incisional biopsy (4 months following the first) was taken from the overlying mucosa of the mandibular ridge. This biopsy showed heavily inflamed connective tissue with prominent exuberant granulation tissue. There was no dysplasia or malignancy identified. The overall findings were granulation tissue with inflammation.

A magnetic resonance imaging (MRI) scan was also requested following the second biopsy. The MRI scan found no abnormal signal at the resection/reconstruction site, and there were no enlarged lymph nodes. The radiologist concluded that there was no convincing MRI evidence for disease recurrence.

### Treatment

Advice was sought from specialists in oral medicine. It was concluded that the proliferative growth was induced by the patient’s use of amlodipine. The patient’s general medical practitioner was informed and asked to change the patient’s antihypertensive medication. It was then arranged for the remaining enlarged soft tissue mass to be excised under local anaesthetic by the maxillofacial surgeon.

### Outcome

**Fig. 5:**
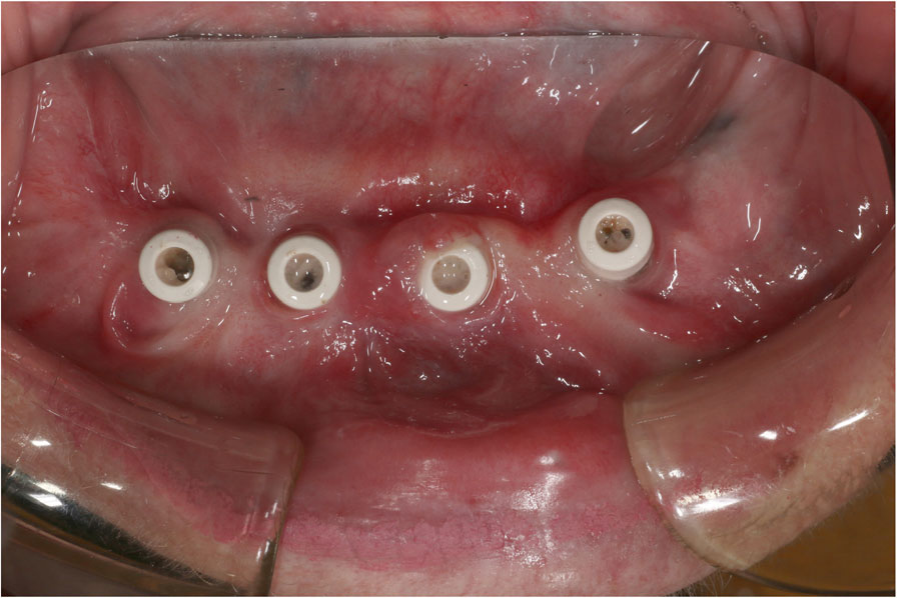
Resolution of gingival enlargement around healing abutments

The growth was excised uneventfully and without reoccurrence. Implant treatment was recommenced shortly after. The overgrown tissue was removed as it was obstructive for the patient and reduced her ability to undertake adequate oral hygiene around the dental implants. There was an expectation that non-surgical peri-implant therapy would be required, but due to the complete resolution of the gingival overgrowth after excision and alteration of her medication, this was not required. The patient required multiple appointments of oral hygiene instruction to allow the healing abutments to become visible and useable (Fig. 5).

**Fig. 6:**
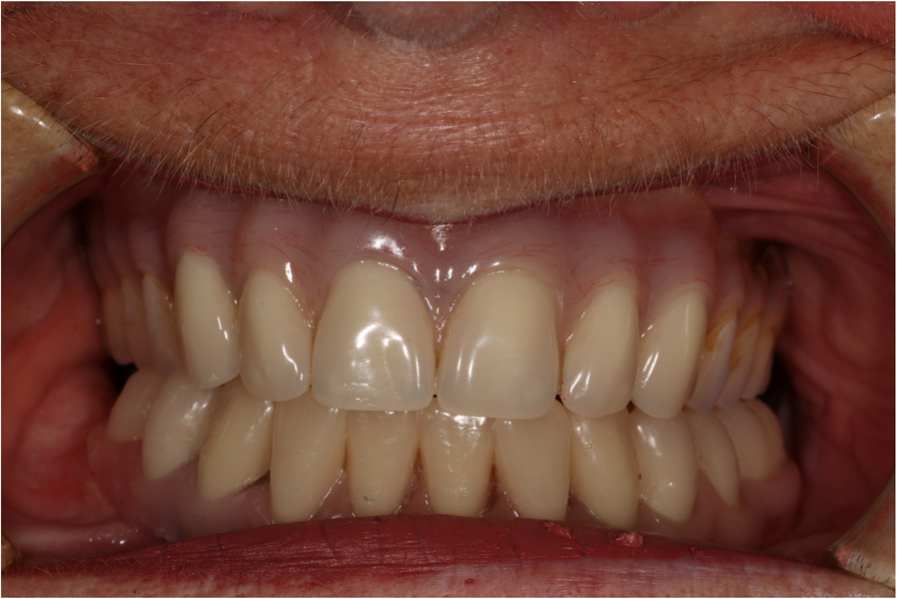
Mandibular implant-retained wax try-in stage and pre-existing maxillary complete denture

**Fig. 7:**
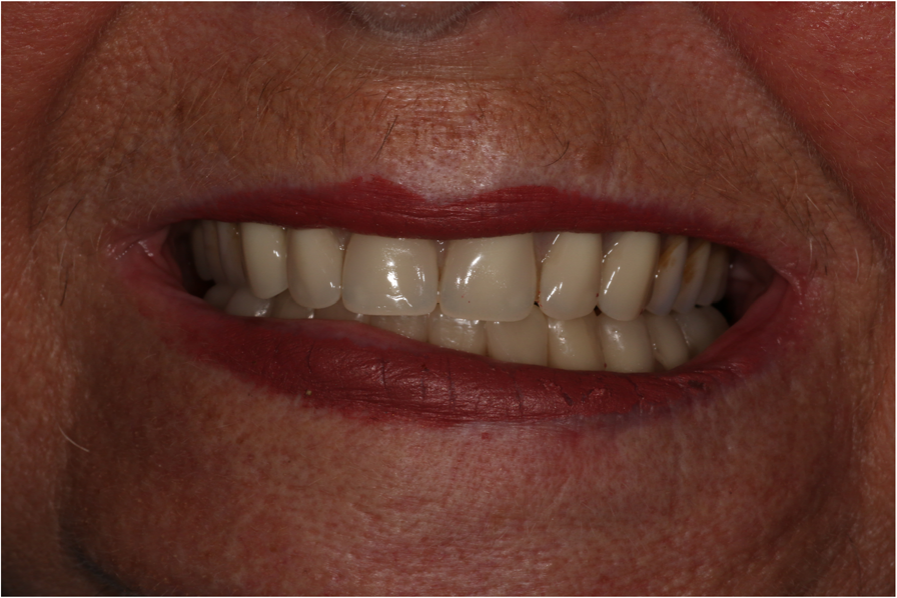
Wax try-in stage showing insufficient lip support and tooth display

At the implant-retained wax rim and wax try-in stage, the occlusion was initially prescribed as a class 1 incisal relationship with bilateral buccal overjets (Fig. 6). However, this did not provide sufficient lower lip support and tooth display for the patient to be satisfied, especially on her right hand side (Fig. 7). This tooth position was also uncomfortable lingually for the patient due to a reduced tongue space.

**Fig. 8:**
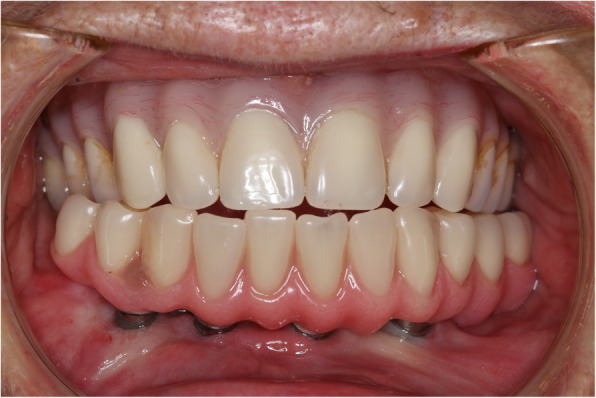
Definitive implant-retained mandibular denture and pre-existing complete denture showing new prescribed occlusion

Thus, the patient and dentist agreed to accept an altered occlusion. The new prescribed occlusion was balanced with simultaneous contacts anteriorly and posteriorly and mild lingual imbrication to provide the patient a more natural appearance (Fig. 8). This additional lip support was also pleasing to the patient.

## Discussion

It is thought that CCBs limit the production of active collagenase leading to a reduction in collagen degradation and causes an increase in collagen accumulation [9]. Other pathways suggest that pro-inflammatory cytokines have an enhancing effect on gingival fibroblasts leading to increased collagen synthesis [11]. CCBs also cause elevated levels of androgens such as testosterone which may act on the gingival cells to cause overgrowth [12].

Amlodipine is less commonly associated with gingival enlargement compared to nifedipine [5]. The prevalence of amlodipine-induced gingival enlargement is 1.7–3.3% compared to nifedipine (14–83%) [2, 3]. Both drugs have a similar structure but nifedipine is highly lipophilic and enters the cell membranes more quickly than amlodipine [8]. Amlodipine also has a higher high life (34 h) than nifedipine (7.5 h) and has a higher volume which means the drug does not circulate in the blood as the drug remains tissue bound and inactive [13].

It is accepted that oral plaque biofilms are a necessary risk factor in CCB-induced gingival enlargement. Enlarged gingival tissue is often confined to dentate areas where the influence of the biofilm exacerbates the effect of the CCB [14]. The placement of an implant may create an area of biofilm formation that was not otherwise present in a previously edentulous patient. Therefore, the implant itself may be a trigger for gingival overgrowth. Alternatively, as described in this case, the gingival enlargement may not manifest until the implants are exposed to the oral environment with the placement of trans-mucosal abutments.

Effective treatment should initially begin with discontinuation of the CCB, after consultation with the general medical practitioner, and switching to an alternative antihypertensive medication class such as angiotensin-converting-enzyme (ACE) inhibitors, diuretics or beta-blockers [15].

Non-surgical periodontal treatment can be effective for mild to moderate gingival enlargement [8]. The mechanical removal of the biofilm can reduce the inflammatory factors that contribute to the disease process [8]. Improved oral hygiene with regular periodontal treatment can help to control milder cases [16]. For moderate to severe cases, surgical treatment is recommended. Excess tissue can be excised (gingivectomy) using scalpels or electrosurgery; however, the latter should not be used around dental implants. However, without alteration to the patient’s medication, recurrence has been reported to occur in up to 40% of patients [17].

Fibula-free flaps are the most commonly used bone-containing free flap in maxillofacial reconstructive surgery [18]. The fibula-free flap provides a consistent bone volume that is suitable for rehabilitation with dental implants [19]. Osseointegration of implants into fibula-free grafts has been shown to be safe and predictable [19, 20]. Gurlek et al. found no significant difference between implants placed in the mandibular bone compared with those in vascularized fibula grafts [21]. However, for patients who have undergone postoperative radiation therapy, there are reduced success rates of implants placed in fibula grafts [22].

The incidence of SCC next to implants is low. It is reported that a history of previous SCC is a risk factor for peri-implant carcinoma. The most common clinical presentation is an exophytic mass around the implant [23]. It is not possible to determine if there is a causal relationship between the presence of implants and the development of SCC around implants [24]. However, studies have shown that SCC is more likely to arise around implants in patients with a previous history of oral cancer [25]. There should be a high level of suspicion for exophytic masses around implants placed for dental rehabilitation in head and neck cancer patients. These masses should be biopsied to exclude SCC recurrence.

## Conclusions

CCB-induced gingival enlargement is a rare presentation in edentulous patients and can be triggered by placement of dental implants to allow for oral rehabilitation or their exposure. This potential complication may be overlooked by dentists and surgeons when informing patients of potential risks. Clinicians should be aware of the presentation of this condition and its management through cessation of the CCB and non-surgical or surgical periodontal treatment if indicated.

## Data Availability

Not applicable.

## Abbreviations

CCB: Calcium channel blocker
MRI: Magnetic resonance imaging
SCC: Squamous cell carcinoma
